# Neuroglobin: A New Player in the Gut–Brain Axis

**DOI:** 10.3390/ijms27146491

**Published:** 2026-07-21

**Authors:** Mohamad Khalil, Laura Mahdi, Ali Madani, Ahmad Arzouni, Elisa Lanza, Camilla Piazzai, Maria Marino, Piero Portincasa

**Affiliations:** 1 Clinica Medica ‘A. Murri’, Department of Precision and Regenerative Medicine and Ionian Area (DiMePre-J), University of Bari ‘Aldo Moro’ Medical School, Piazza Giulio Cesare, 11, 70124 Bari, Italy; laura.mahdi@uniba.it (L.M.); elisa.lanza1992@gmail.com (E.L.); piero.portincasa@uniba.it (P.P.); 2 Rammal Rammal Laboratory, ATAC Group, Faculty of Sciences, Lebanese University, Al-Hadath Campus, Beirut 1003, Lebanon; amadani.ul@gmail.com (A.M.); ahmadarzouni9@gmail.com (A.A.); 3 Department of Science, University of Rome Tre, 00146 Rome, Italy; camilla.piazzai@uniroma3.it (C.P.); maria.marino@uniroma3.it (M.M.); 4 Laboratory of Neuroendocrinology, Metabolism and Neuropharmacology, IRCCS Santa Lucia Foundation, Via del Fosso di Fiorano 64, 00143 Rome, Italy

**Keywords:** neuroglobin, gut–brain axis, microbiota-derived metabolites, neuroprotection, metabolic signaling, estradiol signaling, gut microbiota

## Abstract

Neuroglobin (NGB), initially identified for its oxygen-binding capacity in neuronal tissues, has emerged as a multifunctional protein involved in neuroprotection, oxidative stress regulation, and mitochondrial homeostasis. Although its functions have been extensively studied in the central nervous system (CNS), its potential role in the gut–brain axis (GBA) remains largely unexplored. The GBA integrates neural, endocrine, immune, and metabolic signaling between the gastrointestinal tract and the brain, and growing evidence from microbiome, neurobiology, endocrinology, and nutrition research suggests that several pathways involved in GBA signaling may also influence NGB expression or activity. However, direct experimental evidence, particularly from in vivo studies, remains limited. This hypothesis-driven review integrates evidence from diverse fields to explore the possibility that NGB may represent a molecular link between gut-derived signals and neuroprotective mechanisms. We summarize current knowledge of NGB biology and discuss indirect evidence indicating that microbial metabolites, dietary phytochemicals, and hormonal mediators, including estradiol, may converge on pathways associated with NGB regulation. Based on these observations, we propose a conceptual framework in which NGB could participate in gut–brain communication while emphasizing that this hypothesis requires experimental validation. By bringing together findings that have not previously been considered within a unified context, this review highlights key knowledge gaps, opens new perspectives on the potential involvement of NGB in the GBA, and provides a foundation for future mechanistic studies in neurodegenerative and neuroinflammatory disorders.

## 1. Introduction

Neuroglobin (NGB) was first identified in 2000 [1]. It is the fourth member of the globin family found in humans after erythrocyte hemoglobin (Hb), myoglobin (Mb), and Cytoglobin (Cygb). NGB is a monomeric, oxygen-binding protein predominantly expressed in neurons and retinal cells. Structurally similar to myoglobin, NGB exhibits a bis-histidyl hexacoordinated heme iron, which confers unique ligand-binding properties [2]. Beyond its role in oxygen transport, NGB has been implicated in neuroprotective functions, including the mitigation of oxidative stress and the preservation of mitochondrial integrity [3]. Elevated levels of NGB have been associated with enhanced neuronal survival under hypoxic and ischemic conditions, suggesting its protective role in neurodegenerative diseases such as Alzheimer’s disease [4].

Concurrently, the gut–brain axis (GBA) has emerged as a critical bidirectional communication network linking the gastrointestinal tract and the central nervous system [5]. This complex system encompasses neural, hormonal, and immunological pathways, with the gut microbiota playing a pivotal role in signaling brain function and behavior [6]. Either qualitative, quantitative, or topographic changes in gut microbiota, a condition occurring in gut dysbiosis, have been associated with various neurological and psychiatric disorders, including depression, anxiety, and neurodegenerative diseases [7]. Extensively published research has shown the independent roles of NGB as a neuroprotectant and the GBA as a critical bidirectional communication network. However, a clear knowledge gap persists regarding how NGB functions at the intersection of these two systems, particularly concerning the intestinal barrier integrity and the neuronal signaling along the axis. To address this gap, this review provides a novel, integrated framework focusing on how microbial metabolites and gut-derived hormonal signals modulate NGB expression and function. In doing so, we explicitly distinguish between established, experimentally verified data, and our proposed hypothesis regarding NGB’s potential role as a systemic biomarker for GBA barrier breakdown. This novel perspective aims to provide a balanced foundation for directing future mechanistic and clinical evaluations.

### Literature Search Strategy

To ensure a comprehensive, transparent, and unbiased overview of the literature, a non-systematic literature search was conducted across PubMed/MEDLINE, SCOPUS, and Google Scholar databases. The search spanned from database inception to May 2026, restricting results to peer-reviewed articles, original clinical articles, and authoritative open-access biomedical databases (specifically, the Human Protein Atlas) published in the English language. Search queries utilized combinations of MeSH terms and free-text keywords: (“Neuroglobin” OR “NGB”) AND (“gut–brain axis” OR “gastrointestinal barrier” OR “blood–brain barrier” OR “enteric nervous system”).

Beyond literature retrieval, spatial and structural protein data for human neuroglobin were obtained from the RCSB Protein Data Bank (PDB; Structure ID: 4MPM) and visualized using UCSF ChimeraX 1.11 software (University of California, San Francisco, CA, USA). Schematic diagrams and pathway illustrations mapping the molecular intersections along the GBA were constructed using BioRender.com.

## 2. Genetic, Biochemical, and Functional Profile of NGB

### 2.1. NGB Gene

The human NGB gene (*NGB*) is located on chromosome 14q24.3 and spans approximately 8041 base pairs. Its coding sequence extends from nucleotide 376 to 828 within a 1885 bp transcript. Its promoter region is GC-rich and contains two CpG islands, indicating potential regulation via DNA methylation. Unlike many human gene promoters, it lacks a TATA box, a characteristic found in about 24% of human promoters [8,9].

Several conserved transcription factor-binding sites have been identified within the *NGB* promoter, including two GC-boxes and two neuron-restrictive silencer elements (*NRSEs*). Transcription factors Sp1 and Sp3 are known to bind to these GC-boxes, enhancing NGB expression. The *NRSEs* are potential binding sites for the neuron-restrictive silencer factor (*NRSF*), which may contribute to the neuron-specific expression pattern of NGB [9]. An enhancer region within the first intron of *NGB* has been identified, which is responsive to 17β-estradiol (E2) [10]. E2 induces the binding of estrogen receptor α (ERα) to this enhancer, leading to epigenetic modifications that promote *NGB* transcription. These modifications include increased levels of histone H3 lysine 4 monomethylation (H3K4me1) and histone H3 lysine 27 acetylation (H3K27Ac), markers associated with active enhancers [10].

### 2.2. NGB Protein

**Figure 1:**
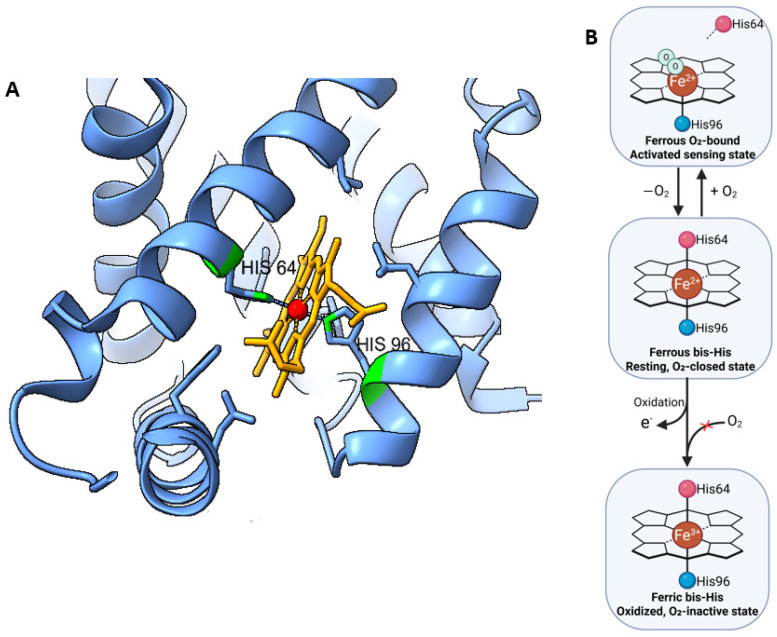
The structural aspects of neuroglobin. (**A**) The 3D structure of NGB (https://www.rcsb.org/structure/4MPM accessed on 12 January 2026) obtained on ChimeraX 1.11 software: ferrous in red; heme group in yellow; the proximal histidine (His96, F8) and the distal histidine (His64, E7) residues in green; NGB protein in blue. (**B**) An illustration of the ferrous and ferric forms of NGB, showing the reversible binding of O_2_ to the ferrous iron, displacing the distal histidine ligand (bis-His conformation), as well as oxidation to the ferric state [12].

NGB is a 151-amino-acid intracellular protein with a molecular weight of 16.9 kDa. It encompasses eight α-helices that surround a heme group in the ferrous (Fe^2+^) or ferric (Fe^3+^) state (Figure 1A). The structure shows a bis-histidyl hexacoordinated configuration, with proximal histidine (HisF8) bound on one side, and the distal histidine (HisE7) bound on the other side under ligand-free conditions [11,12]. An internal hydrophobic pocket and tunnel system connect the protein surface to the active site, facilitating ligand migration. Crucially, human NGB features an internal disulfide bridge (Cys46-Cys55) within its CD-loop. The oxidation state of this bridge induces conformational sliding of the heme, directly altering its ligand affinity based on the surrounding cellular redox environment [13].

### 2.3. Ligand Properties

The ligand-binding properties of NGB are depicted in Table 1. Several features are reported, including interactions with small diatomic molecules such as oxygen (O_2_), nitric oxide (NO), and carbon monoxide (CO), as well as with inorganic ligands such as cyanide (CN^−^) and azide (N_3_^−^). These data highlight the interaction mechanics and the pathophysiological role of these ligands, emphasizing the high affinity of NGB and rapid binding kinetics for both O_2_ and NO, the relative stability of its heme-Fe (II) center, and the modulation of ligand reactivity by structural features such as the Cys (CD5)46–Cys (D5)55 disulfide bond. In particular, the comparable bimolecular rate constants for O_2_ and NO binding emphasize the fine-tuned reactivity of NGB under physiological and hypoxic conditions.

### 2.4. Enzymatic Activity

The (pseudo-)enzymatic activities associated with human NGB, including NO and O_2_ scavenging, nitrite reduction, hydroxylamine reduction, free radical scavenging, and nitration of aromatic substrates, are reported in Table 2. For each activity, we describe the reaction type, relevant intermediates (e.g., NGB(III)-OONO, NGB(III)-NO), kinetic parameters such as rate constants, and the molecular species involved. The human NGB(II)-O_2_ rapidly scavenges NO via a peroxynitrite intermediate [19,27], while NGB(II)-NO can scavenge O_2_ under certain conditions, albeit with low physiological relevance [28]. NGB (II) also displays nitrite reductase activity, influenced by the redox state of Cys residues and the accessibility of the heme pocket [29,30]. Additionally, NGB(III) acts as an effective scavenger of various free radicals [31,32].

## 3. NGB Expression

### 3.1. Physiological Expression

**Figure 2:**
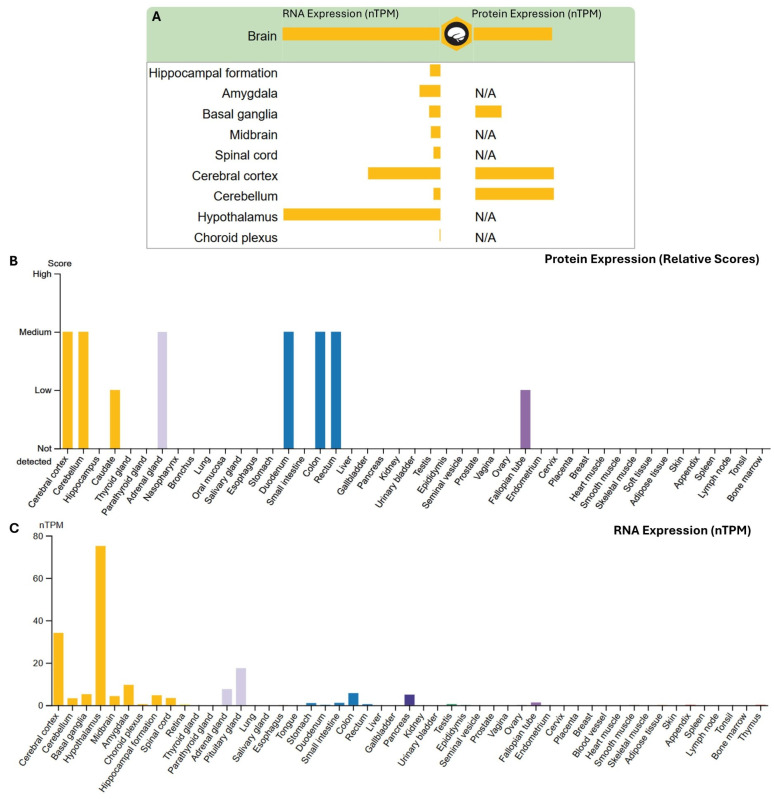
The tissue expression profile of the NGB protein across various human tissues [33]. (**A**) Regional expression of NGB in the central nervous system. The chart displays relative expression levels across various brain and spinal cord regions. (**B**) Protein expression scores of NGB across normal tissues. A broad panel illustrating categorical expression scores (high, medium, low, not detected) showing localized expression in brain and gastrointestinal tract tissues. (**C**) mRNA expression of NGB scores across normal tissues. A broad panel with scores in normalized protein-coding transcripts per million (nTPM).

Based on the Human Protein Atlas (HPA) [33], NGB shows the highest levels of expression in the neurons of the brain, with the highest RNA expression detected in hypothalamus (regulates autonomic, hormonal, and homeostatic functions, and is closely linked to emotional and stress responses), followed by the cerebral cortex (functions in deep thought, planning, and awareness), the amygdala (emotional processing), the basal ganglia, with the caudate (drives routine behaviors and automatic goal-seeking), the hippocampus (memory-related functions), the midbrain, the spinal cord, the cerebellum, and choroid plexus [33] (Figure 2). At the protein level, expression is detectable at a medium level in the cerebral cortex and cerebellum, and at a low level in the caudate [33]. The broad expression across regions tied to emotional regulation, executive function, and habitual behavior suggests a potential influence of NGB on these mental and psychological processes.

NGB is also detectable in the endocrine tissue (adrenal gland), gastrointestinal tract tissue (duodenum, colon, rectum), and female tissue (fallopian tube), both at the RNA and protein levels [34]. Lower RNA expression without corresponding protein detection has been reported in the pancreas (Figure 2). NGB’s RNA expression is enhanced in the glial cells and T cells of the brain, thymus, eyes, adrenal cells, and oocytes. The RNA can also be detected at a slight level in pituitary endocrine cells. Similarly in the ciliated cells of the brain, lung, epididymis, and fallopian tube, in addition to the thymic barrier epithelial cells, lung glandular epithelial cells, germ cells of the testis, brain endothelial cells, mural cells of the brain and adipose tissue, mast cells and adipocytes of the adipose tissue, mononuclear phagocytes of the brain and adipose tissue, and B cells of the kidney ([33], Figure 2).

Functionally, the localized expression of NGB within these gastrointestinal tissues, specifically inside enteric neurons, as well as the mucosal epithelium, serves as a critical endogenous line of defense against localized ischemic and inflammatory insults. Under conditions of intestinal ischemia or active colitis, mucosal hypoxia induces a local NGB upregulation via Hypoxia-Inducible Factor-1 alpha (HIF-1α) pathways. Once elevated, intestinal NGB acts as an intracellular antioxidant, scavenging ROS and RNS for the sake of preventing metabolic stress and ischemic injury [35]. By limiting the intracellular ROS accumulation, NGB prevents the downregulation and structural disruption of essential tight junction proteins, such as Occludins and ZO-1, preserving the intestinal permeability boundaries and preventing systemic endotoxemia [36].

During localized gut inflammation, the inducible nitric oxide synthase (iNOS) generates toxic levels of nitric oxide (NO), and local NGB detoxifies excess NO via the NO-dioxygenase reaction [27] and inhibits the nuclear translocation of NF-kB. This dual action suppresses downstream pro-inflammatory cascades (TNF-α, IL-1β, and IL-6), shielding both the enteric nervous system and the epithelial barrier from structural breakdown and secondary systemic endotoxemia.

NGB protein is normally expressed at low to moderate levels; it is an inducible protein whose levels can increase in response to stimuli such as light exposure, sleep deprivation, and aging. Changes in the central nervous system likely imply effects of NGB on circadian rhythms, neurodevelopment, and neurodegeneration (Table 3).

Hormones such as vascular endothelial growth factor (VEGF), erythropoietin (EPO), thyroid hormones (THs), and 17β-estradiol (E2) strongly upregulate NGB expression through distinct signaling pathways. Crucially, experimental knockdowns or antisense blockades of NGB during 17β-estradiol or hypoxic stress treatments blunt these neuroprotective effects, confirming that NGB serves as an indispensable downstream effector of these survival pathways [10,44,45,46,47,48,49].

In neuronal-derived cells, resveratrol (RSV), a known estrogen receptor β (ERβ) ligand, activates the ERβ/NGB pathway, leading to a rapid and sustained accumulation of NGB in the cytosol and mitochondria. This upregulation enhances mitochondrial function and protects neurons from oxidative stress-induced apoptosis, mirroring the protective mechanism previously observed with 17β-estradiol [50]. Naringenin (Nar), a flavonoid, enhances the neuroglobin (NGB) expression in SK-N-BE cells by interacting specifically with the estrogen receptor β (ERβ) [49]

A panel of natural compounds was screened using mouse and human cell-based reporter systems to identify new NGB inducers [51]. Among these, daidzein (Dzn), genistein, polydatin, and biochanin A significantly increased NGB mRNA expression in both human and mouse primary neurons. Additionally, the study highlights that formononetin-induced NGB upregulation requires activation of the cAMP response element-binding protein (CREB), a transcription factor that is also modulated by gut-derived metabolites such as short-chain fatty acids and neurotransmitter-like compounds. Four of these (excluding polydatin) are phytoestrogens, compounds structurally and functionally like E2. Among them, formononetin showed the strongest induction of NGB, and further studies suggested it acts through activation of the CREB pathway.

**Figure 3:**
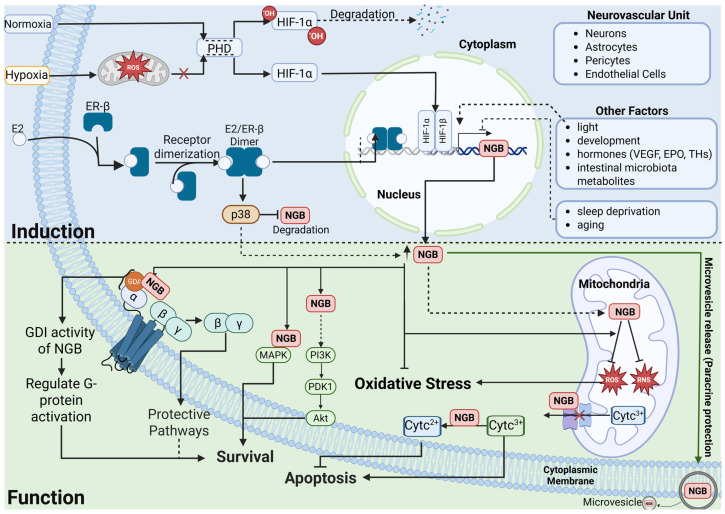
The molecular mechanisms of NGB induction and its protective role against oxidative and mitochondrial stress in the neurovascular unit (NVU) under normoxic and hypoxic stress. Under normoxia, PHD hydroxylates HIF-1α, targeting it for proteasomal degradation. Under hypoxia, PHD activity is inhibited, allowing HIF-1α to translocate to the nucleus and drive NGB transcription. E_2_ binds ER-β, triggering two pathways. In the genomic pathway, the E_2_/ER-β dimer translocates to the nucleus to induce NGB expression. Simultaneously, a non-genomic pathway activates p38 MAPK, which inhibits NGB degradation, facilitating rapid cytosolic accumulation (within 1 h) and promoting its translocation to the mitochondria. Additionally, NGB levels are also influenced by the circadian rhythm and intestinal metabolites. In the functional mechanisms, NGB acts as a GDI for Gαi subunits, regulating G-protein activation and triggering pro-survival signaling via the PI3K/Akt and MAPK pathways. NGB translocates to the mitochondria to scavenge ROS/RNS, mitigating oxidative stress. NGB prevents the apoptotic cascade by binding to ferricytochrome Cytc^3+,^ inhibiting its reduction and the subsequent activation of pro-apoptotic factors. E_2_ facilitates the export of NGB via microvesicles, which are released into the extracellular space to enhance the stress resistance of adjacent neurons. Abbreviations: PHD: Prolyl Hydroxylase; HIF: Hypoxia-Inducible Factor; E2: Estrogen 2; ER: Estrogen Receptor; VEGF: Vascular Endothelial Growth Factor; EPO: Erythropoietin; THs: Thyroid Hormones; GDF: Guanosine Diphosphate; Cytc: Cytochrome C; ROS: Reactive Oxygen Species; RNA: Reactive Nitrogen Species; PI3K: Phosphoinositide 3-Kinase; PDK1: 3-Phosphoinositide-Dependent Protein Kinase-1; Akt: Protein Kinase B; MAPK: Mitogen-Activated Protein Kinase. Created in https://BioRender.com (accessed on 2 July 2026).

Under conditions of hypoxia and ischemia, NGB expression is upregulated by the Hypoxia-Inducible Factor-1 (HIF-1) mechanism [35]. Stabilized HIF-1α then translocates into the nucleus, where it dimerizes with HIF-1β (ARNT) to form the active HIF-1 transcription factor complex. This complex binds to distal hypoxia-responsive elements (HREs) located ~20–30 kb upstream of the *NGB* gene. In addition to its direct enhancer binding, HIF activates several downstream transcription factors, including Sp1, NF-κB, and Egr1, which bind directly to the NGB promoter and synergize to amplify transcription. The enhancer activation (via HIF-1) and promoter activation (via Sp1/NF-κB/Egr1) result in elevated NGB mRNA levels and increased protein synthesis (Figure 3) [52].

At the cellular level, ROS-activated pathways are further enhanced by estrogen receptors β (ERβ). ERβ not only activates the p38 MAPK and PI3K/Akt pathways, leading eventually to cellular survival, but also enhances the translocation of NGB into the mitochondria, where it scavenges ROSs and reactive nitrogen species (RNSs) and binds to cytochrome C, reducing it from cytochrome C^3+^ to cytochrome C^2+^. This step preserves mitochondrial integrity and suppresses apoptotic signals. NGB in mitochondria also binds cytochrome C transporters in the outer mitochondrial membrane and reduces cytochrome C escape into the cytoplasm [53,54,55] (Figure 3) NGB also exhibits a Guanine nucleotide Dissociation Inhibitor state (GDI state), which allows it to bind G-alpha proteins and prevents GDP dissociation from the G-alpha subunit and the ultimate binding of GTP. This inhibits the dangerous signaling imbalance and regulates it [56] (Figure 3).

### 3.2. Role of NGB in Disease

NGB is upregulated under hypoxic, ischemic, and oxidative stress, highlighting its critical neuroprotective role across the neurovascular unit (NVU). While traditionally characterized in neurons, this protective machinery is actively shared by astrocytes, pericytes, and brain microvascular endothelial cells to maintain cellular resilience [10,44,45,46,47,48,49,57]. In CNS diseases, such as stroke, traumatic brain injury, and neurodegenerative disorders like Alzheimer’s (AD) and Parkinson’s (PD), increased NGB levels are consistently associated with reduced neuronal damage and improved outcomes. NGB protects neurons from apoptosis, a key feature of AD pathology, by reacting with cytochrome c released from mitochondria and preventing cell death [58]. It also decreases the neurotoxic activity of amyloid-β, a hallmark of AD, by inhibiting caspase activity through activation of the PI3K/Akt signaling pathway, thereby reducing mitochondrial dysfunction and neuronal apoptosis ([59]). NGB has a role in reducing Tau hyperphosphorylation, which is another hallmark of AD, by inhibiting the glycogen synthase kinase-3β [60]. NGB is also found in the substantia nigra, which is a critical area affected in PD. This location suggests a role for NGB in protecting the dopaminergic neurons from oxidative stress and apoptosis, which are central to PD pathology [61]. An adaptive response of NGB in HD has also been reported, suggesting that NGB may exert neuroprotective effects in HD [62].

Animal models suggest that NGB overexpression leads to smaller infarct volumes and enhanced functional recovery post ischemia. NGB is also inducible by stress-related pathways, including HIF-1α and NF-κB, suggesting a regulatory link between cellular stress responses and NGB expression.

NGB has been implicated in the cellular response to hypoxic–ischemic injury in the brain. Experimental and human studies have shown that NGB expression is upregulated in ischemic brain tissue, particularly in the cortical peri-infarct region following ischemic stroke, suggesting a role in endogenous neuroprotective mechanisms against hypoxia and oxidative stress [63]. In addition to local tissue changes, circulating NGB levels increase during the acute phase of ischemic stroke and have been reported to correlate with markers of disease severity. For example, peak serum NGB concentrations measured within the first days after stroke onset show significant positive correlations with both infarct volume and neurological deficit as assessed by the National Institutes of Health Stroke Scale (NIHSS) [64,65].

The blood–brain barrier (BBB) is protected from leakage during ischemic events due to the expression of NGB in pericytes, which are crucial for barrier maintenance [57]. NGB is overexpressed in brain trauma cases, where it is found to be at its highest levels in the injured neurons, and its peaking depends on the severity of the injury. It scavenges the ROS and RNS that are elevated during oxidative stress directly after a brain trauma [66]. Beyond the brain, NGB is being explored in cancer biology due to its inducibility under metabolic stress, hinting at a broader role in cellular defense. Collectively, the data underscore NGB as a promising therapeutic target for ischemic, hypoxic, and degenerative diseases of the nervous system. The physiological relevance of neuroglobin extends across a diverse spectrum of acute and chronic pathological conditions, where it operates via distinct cell survival pathways (Table 4).

**Figure 4:**
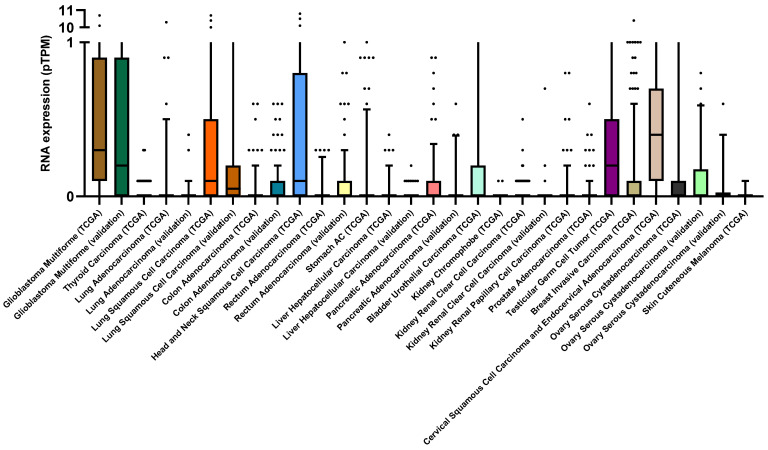
RNA expression of NGB in different cancer cell lines in protein-coding Transcripts Per Million (pTPM) [68]. Because expression is markedly higher in cervical squamous cell carcinoma and endocervical adenocarcinoma than in most other tumor types, differences among cancers with low expression (<0.5 pTPM) are visually compressed.

Beyond neurology, NGB has also been implicated in tumor suppression. Analysis of TCGA datasets revealed that cervical squamous cell carcinoma and endocervical adenocarcinoma exhibited the highest NGB mRNA expression, whereas glioblastoma multiforme, lung squamous cell carcinoma, head and neck squamous cell carcinoma, testicular germ cell tumors, and breast invasive carcinoma showed comparatively lower expression levels (Figure 4). Most other tumor types exhibit median expression below 0.1 pTPM, and are shown to be compressed on the graph, when compared to the significantly higher expression in cervical squamous cell carcinoma and endocervical adenocarcinoma (~2.3 pTPM).

Indeed, NGB plays a dual role. On the one hand, it functions as a compensatory protein against oxidative stress, balancing ROS and protecting against oxidative damage, which is crucial for cancer cell survival and proliferation. In breast cancer, for example, NGB is released into the tumor microenvironment under oxidative stress conditions, and it functions as an autocrine or paracrine factor to enhance cell resilience against oxidative stress and chemotherapy-induced cell death. In addition to the anti-apoptotic properties of NGB, especially in estrogenic receptor α-positive cancer cells, where it is upregulated by E2 to impair the activation of pro-apoptotic pathways, thereby promoting cell survival [67].

On the other hand, in colorectal cancer, NGB functions as a tumor suppressor that is frequently silenced through promoter hypermethylation. NGB downregulation is particularly associated with liver metastasis, and restoration of NGB expression induces G2/M phase arrest, apoptosis, and suppresses proliferation, migration, and invasion in vitro, while inhibiting tumor growth and angiogenesis in vivo [34]. Mechanistically, NGB exerts its tumor-suppressive effects by destabilizing GPR35, a G protein-coupled receptor that drives angiogenesis in the microenvironment. The consistent epigenetic silencing of NGB in CRC positions it as a promising methylation biomarker for early diagnosis, prognosis assessment, and cancer risk evaluation, with potential therapeutic implications for demethylation-based interventions targeting the NGB-GPR35–angiogenesis axis [34]. 

## 4. The Gut–Brain Axis: Cellular and Clinical Overview

The GBA comprises a complex network of bidirectional interactions between the central nervous system (CNS) and the enteric nervous system (ENS). This axis integrates several anatomical and functional components: the CNS (notably the brain and the spinal cord), which processes visceral inputs and executes top-down cognitive and emotional control, the intrinsic ENS (often termed as the “second brain”), embedded within the lining of the gastrointestinal tract to locally govern motility, secretions, and blood flow, and the autonomic nervous system (ANS) [69,70,71]. The ANS serves as the primary physical conduit linking the utilizing sympathetic pathways to modulate gut function during stress and the parasympathetic pathways by the vagus nerve to sustain baseline homeostasis. Together with the neuroendocrine and neuroimmune systems and the gut microbiome, these neural structures establish a dynamic communication pathway translating the complex environmental and luminal signals into systemic physiological responses, establishing a dynamic bidirectional communication pathway that integrates neural, hormonal, and immunological signals between the gastrointestinal tract (GI) and the CNS [70,72]. Alterations of this interconnected system likely drive gastrointestinal changes underlying the symptoms of diseases of gut–brain interaction (DGBIs), formerly functional GI disorders, such as irritable bowel syndrome (IBS) [73]. A key element in this signaling network is the hypothalamic–pituitary–adrenal (HPA) axis. This neuroendocrine system maintains homeostasis and mediates the physiological stress response. Upon exposure to stressors, the hypothalamus secretes corticotropin-releasing hormone (CRH), which stimulates the anterior pituitary to produce adrenocorticotropic hormone (ACTH). ACTH then prompts the adrenal glands to secrete cortisol, the principal stress hormone. Individuals with IBS frequently exhibit increased cortisol levels, along with elevated concentrations of pro-inflammatory cytokines such as interleukin-6 (IL-6) and interleukin-8 (IL-8), and neurotransmitters including norepinephrine and serotonin [5]. Disruption of the HPA axis through these mechanisms is thought to contribute significantly to the pathogenesis of IBS [74].

### Barriers of the Gut–Brain Axis

Barriers within the GBA are a complex system of highly interconnected, morpho-functional components. The mucosal surface of the GI tract represents a primary habitat for diverse microbial communities. These microorganisms reside predominantly within the mucus layer that overlays the epithelium, where they remain spatially separated from intestinal epithelial cells under physiological conditions [75]. This mucus barrier, therefore, functions as a critical interface that both accommodates commensal microbes and limits direct bacterial contact with host tissues, while still allowing exposure to microbial metabolites and other bacterial products [75]. In addition to its crucial role in immune defense, the gut is responsible for nutrient absorption and protects against various pathogens. The intestinal barrier is an integral part of the gut–liver–brain axis, a sophisticated system defined by the two-way communication between the microbiome, the gut, the portal vein, the liver, the biliary tract, the systemic circulation, the brain, and various mediators within the body [75]. This system is dynamic and resilient, and the mechanisms that sustain the GBA barriers are essential for maintaining metabolic balance in both health and disease. Therefore, the function of the intestinal barrier and the preservation of gut homeostasis depend on the structural and functional integrity of the microbiome, mucus, enterocytes, immune system, and gut vascular barrier. To maintain homeostasis along the GBA, an interconnected network of physical, immunological, and vascular barriers regulates the translocation of signaling molecules and protects the CNS (Table 5). The brain barrier in the GBA acts as the final regulatory interface between the CNS and the gut microbiome. It integrates signals from the gut to the brain and vice versa, influencing behavior, immunity, and physiology. It regulates communication between the brain and gut through neural, immune, and endocrine signaling, with a focus on maintaining brain health and systemic balance. Dysfunction of the blood–brain barrier can lead to neurological or psychiatric disorders (e.g., depression, anxiety) [76].

## 5. Potential Intersections Between NGB and the Gut–Brain Axis

### 5.1. Role of Microbiota on NGB Expression

Human studies [90] on 2218 twins with higher visceral fat mass (VFM) suggest a novel connection between the food–microbiota axis and adipose tissue function via the metabolite hippurate and its association with NGB expression. Hippurate, a mammalian–microbial co-metabolite derived from the microbial metabolism of dietary polyphenols into benzoic acid and subsequent hepatic glycine conjugation [91,92], has emerged as a key biomarker linking higher intakes of fruits and whole grains to reduced VFM. Elevated circulating levels of hippurate were associated with increased expression of NGB in adipose tissue, where NGB may function as a local metabolic buffer against oxidative stress and lipotoxicity. By safeguarding adipocyte mitochondrial health, it maintains a balanced adipokine profile and suppresses pro-inflammatory cytokines. This reduction in systemic low-grade inflammation prevents BBB breakdown, thereby indirectly supporting central neuroprotection. However, whether hippurate directly modulates NGB expression within the CNS or the ENS remains unknown, as direct empirical data in neuronal tissues are currently lacking. While NGB has been primarily studied in neuronal and endocrine tissues, its presence in adipose tissue may represent a novel mechanism by which the food–microbiota axis influences overall neurological and systemic metabolic health.

The upregulation of NGB in response to Hippurate may reflect the role of polyphenol-derived metabolites in modulating host–microbiota interactions and the role of NGB in GBA. For example, oral administration of pure resveratrol (RSV) to female Wistar rats (50 and 250 mg/kg) revealed significant alterations in both urinary and fecal metabolomes [93]. Notably, high-dose RSV intake was associated with changes in gut microbiota co-metabolites, including elevated levels of hippurate. These findings suggest that RSV modulates microbial composition and function, influencing host metabolism via the gut–microbiota–liver–kidney axis. The observed increase in Hippurate further supports its role as a key microbial–host co-metabolite in response to polyphenol intake. This behavior points to the potential role of NGB in the relationship between diet, microbial activity, and host metabolic regulation, including pathways implicated in GBA and NGB expression. In another study, cinnamic acid, a polyphenolic compound, upregulated the expression of NGB in HN33 neuronal cells [94]. Microbial metabolites such as SCFAs and tryptophan derivatives may modulate NGB expression through epigenetic and signaling pathways. Butyrate, a SCFA produced by microbial fermentation of dietary fiber [78], acts as a histone deacetylase (HDAC) inhibitor, thereby enhancing histone acetylation at gene promoters and upregulating various neuroprotective genes in neuronal models, including BDNF and genes involved in oxidative stress mitigation [95]. However, in HN33 neuronal cells, NGB expression was slightly upregulated by butyrate (~2.5 fold), but was not evident as the inducible NGB expression with other non-naturally occurring SCFAs, such as valproic acid [94]. Although direct evidence linking butyrate to NGB regulation is lacking, its broad HDAC inhibitory and neuroprotective actions suggest it may epigenetically enhance NGB expression.

Similarly, tryptophan-derived metabolites such as kynurenine modulate neuroinflammation and oxidative stress, pathways known to regulate NGB levels. The kynurenine pathway is activated by inflammatory cytokines and produces neuroactive compounds implicated in mood and neurodegenerative disorders [96]. These metabolites influence major transcriptional regulators and stress response pathways, which are key modulators of NGB expression.

It is crucial to note that SCFA-mediated gene modulation is a multi-step process. SCFAs such as butyrate do not directly trigger NGB transcription; rather, they serve as systemic HDAC inhibitors [95]. So, after a high-fiber diet, SCFAs such as butyrate will be produced by the microbiota; a specific amount will serve as an energy source for the colonocytes, and the rest will exit the colon through the portal vein, reaching the liver. A portion of the butyrate will bypass the liver metabolism and enter the systemic circulation. Since the butyrates are lipophilic, they can cross the brain endothelial cells through the monocarboxylate transporters MCT1 and MCT4 [95]. Then, they enter neurons and glial cells, acting as Class I and II HDACs. This epigenetic priming alters the transcriptional responsiveness of multiple neuronal survival genes, among which NGB represents a downstream beneficiary alongside factors like BDNF. Thus, SCFA exposure represents an indirect permissive mechanism within a complex systemic cascade.

### 5.2. The Role of Gut Microbiota in Estradiol Regulation

Similarly, the pathway connecting gut microbiota to central NGB expression via 17β-estradiol (E2) involves multiple intermediate regulatory steps. 17β-estradiol (E2) enhances NGB expression and mediates its neuroprotective function through both transcriptional and subcellular mechanisms in human neuronal cells [97]. In differentiated human neuronal cell lines (SK-N-BE and NT-2), E2 induces NGB expression through ERα binding to regulatory regions of the NGB locus, including the promoter and a putative intronic enhancer, promoting chromatin remodeling and transcriptional activation [10]. In addition, rapid E2 signaling pathways have been widely reported to contribute to this mechanism. Notably, E2 increases NGB levels within approximately 1 h, a time frame that is too short to be explained exclusively by classical genomic mechanisms [49,54,98]. Functionally, E2 exerts multiple protective actions on the gut microbiome and the gut–brain axis. First, it contributes to the maintenance of intestinal eubiosis by preserving microbial diversity and supporting the abundance of health-associated taxa. Reviews highlight that E2 remodels the gut microbial community and stabilizes microbial metabolites such as short-chain fatty acids and secondary bile acids, which are essential for intestinal and neural homeostasis [99].

Second, E2 plays a key role in strengthening the intestinal barrier. It enhances epithelial defense mechanisms by increasing mucin gene expression and supporting tight-junction integrity, thereby protecting the mucosal surface from inflammatory or ischemic insults. Experimental studies in aged female mice demonstrate that E2 replacement improves colonic epithelial resilience after stroke, promotes mucus production, and is associated with beneficial shifts in gut bacterial composition, particularly *Lactobacillus* and *Bifidobacterium*.

Finally, through its regulatory effects on microbiota composition and epithelial health, E2 supports the gut–brain axis. Its neuroprotective influence involves modulation of microbial metabolites, attenuation of neuroinflammation, and stabilization of synaptic and cognitive functions. Evidence from Alzheimer’s disease research demonstrates that the E2–microbiota–brain axis forms a reciprocal regulatory network in which E2 enhances synaptic plasticity and reduces neuropathology, while the microbiota sustains E2 bioavailability via β-glucuronidase activity. Whether the overexpression of NGB is involved in these protective effects is far from being established [100,101,102]. Beyond its well-established roles in digestion, immunity, and metabolism, the intestinal microbiome has emerged as a key modulator of systemic hormone levels through enzymatic activity, metabolic interactions, and neuroendocrine signaling pathways. This bidirectional communication between the gut and endocrine systems not only affects reproductive physiology but also has implications for mood and mental health [103]. Gut microorganisms influence E2 by direct enzymatic metabolism via microbial-derived steroid-modifying enzymes, and by indirect hormonal modulation through the gut–brain axis, particularly via the hypothalamic–pituitary–gonadal (HPG) and hypothalamic–pituitary–adrenal (HPA) axes [103]. E2 is primarily metabolized in the liver into water-soluble conjugates via sulfation and glucuronidation, which are excreted through the kidneys or bile into the intestines [104]. Certain gut bacteria, through the expression of specific enzymes, can reactivate these conjugated forms, thereby modulating circulating hormone levels [105]. For example, *Eggerthella lenta* strain C592 expresses 17β-hydroxysteroid dehydrogenases (17β-HSD), facilitating the oxidative conversion of active E2 to less active estrone (E1) [106]. *Klebsiella aerogenes*, a bacterium capable of degrading E2, was isolated from the fecal samples of premenopausal women diagnosed with depression [107]. In mice, oral administration of this strain led to E2 decline and depression-like behaviors. The responsible enzyme, 3β-hydroxysteroid dehydrogenase (3β-HSD), conferred estradiol-degrading capacity to *E. coli* upon heterologous expression, similarly reducing E2 and triggering depressive phenotypes in mice [107]. Notably, *Clostridium perfringens* has been identified in the gut microbiota of perimenopausal women with depression, with the bacterium expressing a 3β-hydroxysteroid dehydrogenase (3β-HSD) enzyme capable of degrading E2 [108]. Transgenic *E. coli* expressing 3β-HSD replicated this effect in rats, reducing serum and brain E2 and inducing depression-like behavior. These findings suggest a novel microbiota–hormone–brain axis, implicating microbial E2 metabolism as a potential contributor to mood disorders and highlighting the need for further research into E2-degrading bacteria and enzymes as targets for therapeutic intervention.

Emerging evidence underscores the intricate role of the intestinal microbiota in modulating E2 levels by influencing both the hypothalamic–pituitary–gonadal (HPG) and hypothalamic–pituitary–adrenal (HPA) axes [103]. The HPG axis governs reproductive hormone regulation, with gonadotropin-releasing hormone (GnRH) stimulating the release of follicle-stimulating hormone (FSH) and luteinizing hormone (LH), both of which promote ovarian E2 synthesis. However, microbial endotoxins such as lipopolysaccharides (LPSs) can suppress GnRH and LH secretion, leading to reduced E2 levels [109,110,111].

Thyroid hormones are essential for brain development due to their roles in promoting growth and differentiation. An in vivo study demonstrated that administration of high doses of triiodothyronine (T3) in rats led to increased expression of two brain-expressed globins, i.e., NGB and cytoglobin [47]. Although this study confirmed elevated gene and protein levels of both globins, the specific mechanisms by which T3 exerts this effect remain unclear. One proposed explanation is that T3 may indirectly enhance NGB expression via the activation of hypoxia-inducible factor 1 (HIF-1) [47]. Additionally, erythropoietin has been identified as a neuroprotective factor capable of upregulating NGB expression [46], as reported in the brain of Mongolian gerbils [45].

### 5.3. Phytochemicals

**Figure 5:**
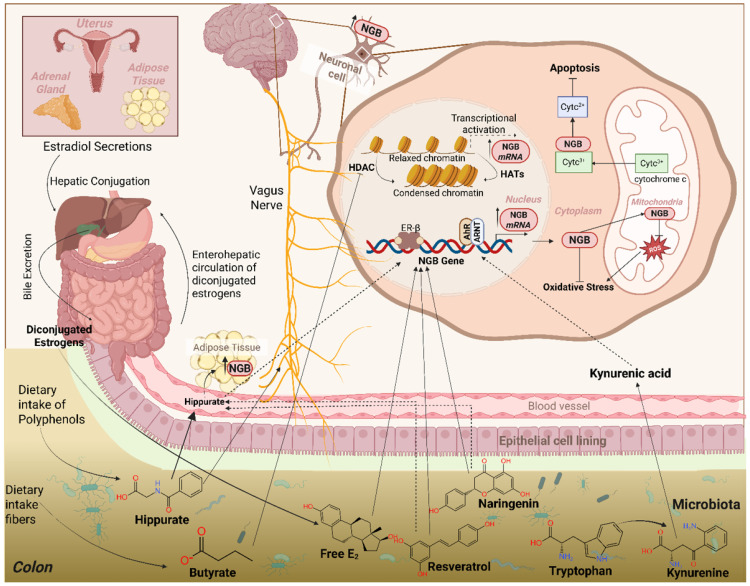
The potential role of NGB in the gut–brain axis. E_2_ is secreted by the uterus, adrenal glands, and adipose tissue. Following hepatic conjugation, deconjugated estrogens undergo enterohepatic circulation via bile secretion. Gut microbiota deconjugates estrogens into free E_2_ and metabolizes dietary fibers and polyphenols into bioactive metabolites, including Butyrate (an HDAC inhibitor), Hippurate, Naringenin, and Resveratrol, in addition to the tryptophan pathway that yields Kynurenic acid, which is a known neuroprotective metabolite. These gut-derived metabolites and free E_2_ reach the brain to modulate NGB expression. This process is governed by ER-β and AhR/ARNT transcriptional complexes, alongside epigenetic remodeling via HAT and HDAC activity. NGB mRNA is exported to the cytoplasm for translation. The resulting NGB protein translocates to the mitochondria to scavenge ROS/RNS and bind to Cytc^3+,^ thereby inhibiting the apoptotic cascade and enhancing neuronal resilience against oxidative stress. Dashed lines indicate proposed or emerging regulatory pathways. Abbreviations: Cytc: Cytochrome C; NGB: Neuroglobin; ROS: Reactive Oxygen Species; HATs: Histone Acetyl Transferases; HDAC: Histone Deacetylase; ER: Estrogen Receptor; AhR: Aryl hydrocarbon Receptor; ARNT: Aryl hydrocarbon Receptor Nuclear Translocator. Created in https://BioRender.com (accessed on 19 February 2026).

Polyphenols represent a broad class of bioactive dietary compounds with well-documented antioxidant, anti-inflammatory, and neuroprotective properties. Increasing evidence indicates that their protective actions extend across the gut–brain axis, where they modulate microbiota composition, reinforce epithelial and blood–brain barrier integrity, and attenuate neuroinflammatory signaling [112]. Among them, resveratrol emerges as one of the most mechanistically characterized molecules. In neuronal cells, resveratrol acts as a ligand of ERβ and activates the same intracellular pathway through which E2 induces NGB expression, leading to a rapid and persistent accumulation of NGB in both cytosol and mitochondria and conferring protection against oxidative stress-induced apoptosis. This ERβ/NGB axis, originally described for E2, is therefore shared by resveratrol, supporting the concept that exogenous polyphenols may mimic selective estrogenic signaling to enhance neuronal resilience. More broadly, the E2-like activities of several polyphenols, including their ability to bind ERβ and modulate endocrine and redox pathways, suggest that diet-derived molecules and endogenous E2 may converge on common molecular nodes, with NGB representing a plausible point of integration. Although direct NGB induction has been experimentally confirmed only for resveratrol, the demonstration that other polyphenols such as naringenin possess ERβ-modulating and neuroprotective properties reinforces the hypothesis of a shared protective architecture in which both endogenous hormones and plant-derived compounds leverage ERβ-dependent mitochondrial stabilization and anti-apoptotic pathways to preserve brain function and gut–brain homeostasis [50,113,114,115,116]. The potential interaction between gut microbiota/metabolites and the expression of NGB is summarized in Figure 5.

## 6. Clinical Relevance and Translational Potential

The wide range of physiological mechanisms of action of NGB can pave the way for additional roles of NGB in several diseases and for potential therapeutic implications within the GBA.

In Alzheimer’s disease, an unhealthy gut was highly linked to neuroinflammation and amyloid-β (Aβ) aggregation, thereby increasing the incidence of disease progression. NGB overexpression in neuronal models reduces Aβ-induced apoptosis by suppressing activation of caspase-3 and caspase-9, an effect mediated by the PI3K/Akt signaling cascade. Akt activation helps protect mitochondrial integrity and maintain energetic homeostasis, thereby preventing neuronal loss. NGB has a role in decreasing tau hyperphosphorylation by inhibiting glycogen synthase kinase-3β (GSK-3β) downstream of Akt, thus reducing tau pathology in Alzheimer’s models [59,117].

Similarly, in Parkinson’s disease, gut dysbiosis may contribute to neurodegenerative cascades. In dopaminergic neuronal models, NGB interacts with mitochondrial complex I to maintain mitochondrial membrane potential, suppresses ROS accumulation, and inhibits caspase-9 activation and apoptosis. By stabilizing the mitochondrial function and reducing the oxidative stress, neurons will [118].

Given these multiple lines of evidence, NGB expression could be enhanced by gene therapy vectors, small molecules that can upregulate it, or modulators that can interfere with the upstream signaling pathway, such as PI3K/Akt activators, and as such, new promising possibilities could be feasible in the world of neurodegenerative therapies.

The bidirectional communication between the gut and the brain is mediated by neural, immune, and endocrine pathways. Dysbiosis can lead to neuroinflammation and neuronal degeneration, paving the way for AD, PD, and other neurodegenerative diseases. NGB plays a critical role in protecting against ROS and apoptosis. Emerging evidence suggests that NGB expression can be indirectly influenced by the gut via SCFAs, such as butyrate. They can cross the blood–brain barrier and modulate gene expression by inhibiting HDACs, thereby influencing NGB expression and neuroinflammatory pathways [119,120,121].

Interventions aimed at restoring the balance in the gut microbiota are promising in their results due to their ability to mitigate the neurodegenerative processes. Fecal microbiota transplantation (FMT), dietary interventions, and the use of probiotics and prebiotics can fix microbial composition and function. And as such, SCFAs are produced together with other metabolites and potentially enhance NGB expression and reduce neuroinflammation [122,123].

The interplay between the gut microbiota and gene expression offers a novel perspective in understanding and managing neurodegenerative diseases. While the precise mechanisms remain to be fully elucidated, targeting the gut microbiota to modulate NGB expression presents a promising therapeutic avenue. Further research is needed to explore this potential and develop effective interventions.

## 7. Current Gaps and Future Directions

The GBA is known for its ability to influence various neurological functions, but its specific role in NGB expression remains largely unexplored. Most studies focus on broader neurological effects rather than specific proteins, such as NGB. Methodological limitations in existing research, such as the absence of functional assessments and translational scope, impede understanding of the role of gut microbiota in NGB expression. Most current studies rely on neuronal cell lines or cross-sectional human data, limiting mechanistic insights.

A critical appraisal of this compiled literature reveals significant translational gaps where many mechanistic data regarding NGB regulation by microbial metabolites (e.g., butyrate) or phytochemicals (e.g., resveratrol) rely exclusively on in vitro cell lines (such as HN33 or SK-N-BE) exposed to supraphysiologic concentrations. These isolated models fail to replicate the complex, multi-layered physical boundaries of the intestinal epithelial barrier or the selective permeability of the blood–brain barrier in vivo. Furthermore, stark biological discrepancies exist regarding the NGB’s dual role in oncology; while it functions as an epigenetic tumor suppressor in colorectal cancer via GPR35 destabilization, it actively promotes cell survival and chemotherapy resistance in breast cancer cells under estrogenic control [34]. It remains entirely speculative whether gut dysbiosis-induced shifts in systemic estradiol bioavailability can truly modulate NGB levels in distant neural tissues to a degree that alters neurodegenerative outcomes. To provide a clear overview of these underlying challenges, Table 6 synthesizes and contrasts verified empirical mechanisms from speculative concepts across the current NGB-GBA literature landscape.

To overcome these limitations and bridge these critical gaps, future research must prioritize a structural pivot away from descriptive associative data toward rigorous in vivo knock-out models to validate these hypothetical intersection nodes.

## 8. Conclusions

Emerging evidence suggests that neuroglobin (NGB) may represent a potential molecular interface linking metabolic, endocrine, and microbial signaling within the gut–brain axis. Although NGB has been extensively investigated for its neuroprotective functions, including the preservation of mitochondrial function and the limitation of oxidative stress, the possibility that its expression is influenced by gut microbiota-derived metabolites, dietary phytochemicals, and hormonal regulators such as E2 is supported primarily by indirect evidence and in vitro studies. Metabolites including hippurate, short-chain fatty acids (SCFAs), and tryptophan derivatives provide examples of microbial products that have been associated with transcriptional and epigenetic pathways relevant to NGB regulation, although direct causal relationships have not yet been demonstrated in vivo.

Collectively, these findings support the hypothesis that NGB could act as an integrative mediator through which dietary patterns, microbial metabolism, and endocrine signals converge to influence the cellular pathways involved in neuronal resilience. This proposed role is particularly relevant to neurodegenerative disorders such as Alzheimer’s and Parkinson’s diseases, in which oxidative stress, mitochondrial dysfunction, and neuroinflammation are key pathological features. However, current evidence is insufficient to conclude that modulation of the gut microbiota or dietary interventions directly regulate NGB expression or activity in vivo, or that such modulation translates into neuroprotection.

Future studies combining microbiome profiling, metabolomics, and mechanistic molecular approaches in appropriate animal models and human cohorts will be essential to establish whether gut microbiota-derived signals directly regulate NGB and to determine the physiological and therapeutic relevance of this proposed gut–microbiota–NGB axis.

## Figures and Tables

**Table 1:** Kinetic Mechanisms and Pathophysiological Roles of Neuroglobin Ligand Binding.

Ligand	Interaction Mecahnics	Pathophysiological Role	References
O_2_	Reversible binding (P_50_ ranges from 0.9 to 10 mmHg), limited by cleavage of the His(E7)64-Fe bond Shows temperature/pH-dependent Bohr effect	Acts as an oxygen sensor during cerebral ischemia and hypoxia.	[14,15,16,17]
NO	High intrinsic affinity inhibited by His(E7)64 competitive inhibition; Binding limited by cleavage of His(E7)64-Fe bond	Scavenges toxic NO during active neuronal/gut inflammation.	[17,18,19,20]
CO	Two-step binding process involving pocket dynamics; strongly affected by local cellular redox states and internal motions	Decreases CO toxicity, improves systemic oxygen delivery, and reverses the mitochondrial inhibition.	[2,14,19,21,22,23,24,25,26]
CN−	Two-step binding process influenced by heme isomerization and pocket residues	Interacts with CN− to buffer mitochondrial toxicity and maintain cellular homeostasis	[26,27,28,29,30]

Abbreviations: O_2_, oxygen; CO, carbon monoxide; CN−, Cyanide; NO, nitric oxide; NGB, neuroglobin; His, histidine; Fe, iron; mmHg, millimeters of mercury.

**Table 2:** Enzymatic properties of human NGB.

Reaction/Activity	Main Reactants	Products	Mechanism/Notes	Rate Constants	References
NO scavenging	Human NGB(II)-O_2_ + NO	NGB(III) + NO_3_^−^	Rapid via NGB(III)-OONO intermediate	λ ≈ 360 s^−1^ (pH 7.0, 20 °C)	[19,27]
O_2_ scavenging	Human NGB(II)-NO + O_2_	NGB(III) + NO_3_^−^	Bi-phasic; slow and unlikely in vivo	h′f = 1.6 × 10^1^ M^−1^s^−1^; h′s = 0.4 M^−1^s^−1^	[19,28]
Oxidation by peroxynitrite	Human NGB(II)-NO + ONOO^−^	NGB(III) + NO	Fast oxidation; mono-exponential decay	h′f = 1.3 × 10^5^ M^−1^s^−1^; λ = 0.12 s^−1^ (pH 7.2, 20 °C)	[28]
Nitrite reduction	Human NGB(II) + NO_2_^−^	NGB(III) + NGB(II)-NO	Redox-sensitive; Cys (CD5)46-Cys(D5)55 bond important	k′f = 6.2 × 10^−2^ to 1.2 × 10^−1^ M^−1^s^−1^ (pH 7.4, 25 °C)	[29,30]
Free radical scavenging	Human NGB(III) + radicals (superoxide, etc.)	NGB(II)-O_2_ (in case of superoxide)	Protective; IC_50_ = 7.4 × 10^−6^ M (superoxide scavenging)	—	[31,32]

Abbreviations: NO, nitric oxide; O_2_, oxygen; NO_3_^−^, nitrate; ONOO^−^, peroxynitrite; NO_2_^−^, nitrite; NGB(II), ferrous neuroglobin; NGB(III), ferric neuroglobin; NGB(III)-OONO, ferric-peroxynitrite intermediate; Cys, cysteine; IC_50_, half maximal inhibitory concentration; λ, observed first-order rate constant; h′f, fast second-order rate constant; h′s, slow second-order rate constant; k′f, forward second-order rate constant; M, molar concentration; M^−1^s^−1^, inverse molar per second; s^−1^, per second; pH, potential of hydrogen.

**Table 3:** Main physiological modulators of NGB expression and its function.

Modulator	Effect on NGB	Experimental System	Mechanism/Notes	References
Light	Increases NGB in SCN neurons (circadian rhythm) and retina (after LED exposure)	Rat brain (SCN, retina); mouse retina	NGB peaks during the light phase; increases after blue, green, and red-light exposure; a marker of retinal damage	[37,38,39]
Sleep deprivation	Decreases NGB-positive cells	Rat brain	Suggests a role in the sleep/wake cycle independent of oxidative stress	[40]
Development	NGB increases during neuronal differentiation	Human ESCs, rat brain, N2a cells	NGB promotes neurite/axonal outgrowth	[41,42]
Aging	NGB declines with age	Rat brain (3–24 months)	Decline in cortex, hippocampus, cerebellum linked to neurodegeneration susceptibility	[43]
Hormones (VEGF, EPO, THs, Estrogens)	Upregulation of NGB expression	Neuronal-derived cells: mouse cerebrocortical neurons, gerbil brain (cerebral cortex and hippocampus), SK-N-BE neuroblastoma cells, primary cortical astrocytes, mouse hippocampal neurons	VEGF stimulates NGB via VEGFR2/Flk1; NGB also suppresses VEGF (feedback loop). EPO upregulates NGB under hypoxia. Thyroid hormones (THs) increase NGB post-treatment and after thyroidectomy. Estrogens (E2) via ERβ and ERα induce NGB expression and mitochondrial localization, enhancing neuroprotection; this involves AKT activation and chromatin remodeling through an intronic enhancer (no canonical ERE present).	[10,44,45,46,47,48,49]

Abbreviations: SCN: Suprachiasmatic Nucleus; LED: Light-Emitting Diode; ESC: Embryonic Stem Cells; NGB: Neuroglobin; N2a: Neuro-2a; VEGF: Vascular Endothelial Growth Factor; EPO: Erythropoietin; THs: Thyroid Hormones.

**Table 4:** Functional roles and molecular mechanisms of NGB in distinct pathological states.

Disease Context	Role of NGB	Mechanism of Action	References
Ischemic Stroke	Neuroprotective, reduces infarct volume, and improves neurological function	Scavenges ROS/RNS, inhibits apoptosis, supports mitochondrial function	[66]
Hypoxia	Enhances neuronal survival	Induced by HIF-1α, NF-κB, and CREB pathways; supports mitochondrial resilience	[35]
Neurodegenerative Diseases	Potential protective role (Alzheimer’s, Parkinson’s, Huntington’s diseases)	Reduces oxidative stress and modulates neuroinflammatory signaling	[58]
Brain Trauma	Reduces tissue damage and neuronal apoptosis	Antioxidant activity, preserves cellular integrity	[64]
Cancer (Emerging)	Dual role (protective or tumor-supportive depending on context)	Induced under metabolic and oxidative stress; links to cell survival	[34,67]

Abbreviations: CREB, cAMP response element-binding protein; HIF-1α, hypoxia-inducible factor 1-alpha; NF-κB, nuclear factor kappa-light-chain-enhancer of activated B cells; NGB, neuroglobin; RNS, reactive nitrogen species; ROS, reactive oxygen species.

**Table 5:** Barriers of the gut–brain axis and representative examples of barrier dysfunction.

Level of Barrier	Main Component	Function/Role	Representative Example of Dysfunction	References
Gut microbiota	Commensal microorganisms	Nutrient metabolism and immune maturation	Depletion of *Faecalibacterium prausnitzii* in Crohn’s disease	[7,77,78,79,80]
Gut mucus (Extracellular Barrier)	MUC2 mucin, goblet cells	Separate microbes from epithelium	MUC2 depletion and mucus thinning in ulcerative colitis	[75,81,82]
Epithelial barrier and tight junctions	Enterocytes, Tight junction proteins (claudin, Occludin, ZO-1)	Controls intestinal permeability	Occludin/claudin downregulation causes increased intestinal permeability	[36,75,83]
Immune barrier	Paneth cells, T cells, B cells, Macrophages	Immune surveillance	Paneth cell dysfunction in Crohn’s disease	[75,84]
Gut–vascular interface	Endothelial cells, Pericytes, Enteric glial cells	Restrict bacterial dissemination	Increased permeability permitting LPS translocation	[75,85]
Liver barrier	Kupffer cells and hepatocytes	Filter gut-derived microbial products	Reduced Kupffer cell clearance leading to endotoxemia in chronic liver disease	[75,86]
Blood–brain barrier	Brain endothelial cells, astrocytes, pericytes	Protects CNS	Increased BBB permeability in Alzheimer’s disease and major depression	[87,88,89]

Abbreviations: BBB, Blood–brain barrier; CNS, Central nervous system; LPS, Lipopolysaccharide; MUC2, Mucin 2; ZO-1, Zonula occludens-1.

**Table 6:** Methodological Uncertainties, Controversies, and Evidence Gaps in NGB-GBA Research.

Focus Area	Established Mechanism	Current Controversy	Methodological Limitation	References
Phytochemical Induction	Resveratrol activates the ERβ/NGB axis to protect against oxidative stress in vitro	Conflicting data on whether dietary polyphenols reach the central nervous system in active concentrations to trigger NGB	Studies heavily rely on treating cell cultures with parent compounds rather than their in vivo hepatic/microbial metabolites	[50]
Short-Chain Fatty Acids (SCFAs)	Butyrate acts as an HDAC inhibitor, broadly upregulating neuroprotective pathways	Butyrate upregulates NGB expression (~2.5-fold) in HN33 cells compared to synthetic pan-HDAC inhibitors	Dependency on static in vitro models; lacks dynamic evaluation under in vivo blood–brain barrier transport restriction	[94]
Oncological Dichotomy	NGB preserves mitochondrial integrity during cellular stress	NGB acts as a tumor promoter in breast cancer but is epigenetically silenced as a tumor suppressor in colorectal cancer	Lack of clinical trials or longitudinal human biomarker data tracking tissue-specific NGB expression variations during cancer progression	[34,124]
Estrogen–microbiome axis	E2 upregulates NGB transcription in neurons via chromatin remodeling	Conflicting evidence on whether gut dysbiosis-mediated shifts in systemic active E2 significantly alter central neuronal NGB concentrations in vivo	Dependency on fecal correlation data	[10,103]
Adipose tissue/hippurate	Hippurate acts as a marker of gut diversity and correlates with elevated NGB in visceral fat mass	It remains entirely hypothetical if adipose tissue NGB can release remote neuroprotective signal proteins to the CNS via the GBA	Total lack of direct empirical evaluation testing hippurate administration on central or enteric neuronal NGB levels	[90]

## Data Availability

No new data were created or analyzed in this study. Data sharing is not applicable to this article.

## Abbreviations

The following abbreviations are used in this manuscript:

17β-HSD	17β-Hydroxysteroid dehydrogenase
ACTH	Adrenocorticotropic hormone
AD	Alzheimer’s disease
AKT	Protein kinase B
ANS	Autonomic nervous system
ARNT	Aryl hydrocarbon receptor nuclear translocator
BBB	Blood–brain barrier
BDNF	Brain-derived neurotrophic factor
cAMP	Cyclic adenosine monophosphate
CN^−^	Cyanide ion
CNS	Central nervous system
CO	Carbon monoxide
CRH	Corticotropin-releasing hormone
CREB	cAMP response element-binding protein
CYGH	Cytoglobin
CYTC	Cytochrome c
DNA	Deoxyribonucleic acid
DZN	Daidzein
ENS	Enteric nervous system
EPO	Erythropoietin
ER	Estrogen receptor
ER α	Estrogen receptor alpha
ER β	Estrogen receptor beta
ESC	Embryonic stem cells
Fe^2+^	Ferrous iron
Fe^3+^	Ferric iron
FMT	Fecal microbiota transplantation
FSH	Follicle-stimulating hormone
GBA	Gut-Barin Axis
GDF	Guanosine Diphosphate
GDI	GDP dissociation inhibitor
GnRH	Gonadotropin-releasing hormone
GPR35	G protein-coupled receptor 35
H3K27ac	Histone H3 lysine 27 acetylation
H3K4me1	Histone H3 lysine 4 monomethylation
HATS	Histone acetyltransferases
HB	Hemoglobin
HD	Huntington’s disease
HDAC	Histone deacetylase
HIF-1	Hypoxia-inducible factor 1
HN33	Hybrid neuronal cell line 33
HPA	Human Protein Atlas
HPG	Hypothalamic–pituitary–gonadal axis
HREs	Hypoxia response elements
HisE7	Histidine at position E7 (distal histidine in globins)
HisF8	Histidine at position F8 (proximal histidine in globins)
LED	Light-emitting diode
LH	Luteinizing hormone
LPS	Lipopolysaccharide
MAPK	Mitogen-activated protein kinase
MB	Myoglobin
N2A	Neuro-2a (mouse neuroblastoma cell line)
N3^−^	Azide ion
NAR	Naringenin
NF-κB	Nuclear factor kappa B
NGB	Neuroglobin
NRSF	Neuron-restrictive silencer factor
NRSEs	Neuron-restrictive silencer elements
NO	Nitric oxide
O_2_	Molecular oxygen
PD	Parkinson’s disease
PDK1	3-Phosphoinositide-dependent protein kinase-1
PI3K	Phosphoinositide 3-kinase
RNA	Ribonucleic acid
RNS	Reactive nitrogen species
ROS	Reactive oxygen species
RSV	Resveratrol
SCFA	Short-chain fatty acids
SCN	Suprachiasmatic nucleus
SP1	Specificity protein 1
SP3	Specificity protein 3
TATA	TATA box (DNA promoter sequence)
THS	Thyroid Hormones
VEGF	Vascular endothelial growth factor
VFM	Visceral fat mass
